# Interventions to Reduce Exposure to Synthetic Phenols and Phthalates from Dietary Intake and Personal Care Products: a Scoping Review

**DOI:** 10.1007/s40572-023-00394-8

**Published:** 2023-03-29

**Authors:** Tiffany C. Yang, Nicolas Jovanovic, Felisha Chong, Meegan Worcester, Amrit K. Sakhi, Cathrine Thomsen, Ronan Garlantézec, Cécile Chevrier, Génon Jensen, Natacha Cingotti, Maribel Casas, Rosemary RC McEachan, Martine Vrijheid, Claire Philippat

**Affiliations:** 1 Bradford Institute for Health Research, Bradford Teaching Hospitals NHS Foundation Trust, Bradford, UK; 2 University Grenoble Alpes, Inserm U1209, CNRS UMR 5309, Team of Environmental Epidemiology Applied to Reproduction and Respiratory Health, Institute for Advanced Biosciences, 38000 Grenoble, France; 3 Norwegian Institute of Public Health, Oslo, Norway; 4 CHU de Rennes, Univ Rennes, Inserm, EHESP, Irset (Institut de Recherche en Santé, Environnement Et Travail) - UMR_S 1085, F-35000 Rennes, France; 5 Health and Environment Alliance (HEAL), Brussels, Belgium; 6 ISGlobal, Barcelona, Spain; 7 Universitat Pompeu Fabra (UPF), Barcelona, Spain; 8 CIBER Epidemiología Y Salud Pública (CIBERESP), Barcelona, Spain

**Keywords:** Scoping review, Endocrine disrupting compounds, Dietary intake, Personal care products, Intervention, Bisphenols, Phthalates, Parabens, Triclosan

## Abstract

**Purpose of Review:**

A scoping review was conducted to identify interventions that successfully alter biomarker concentrations of phenols, glycol ethers, and phthalates resulting from dietary intake and personal care product (PCPs) use.

**Recent Findings:**

Twenty-six interventions in populations ranging from children to older adults were identified; 11 actively removed or replaced products, 9 provided products containing the chemicals being studied, and 6 were education-only based interventions. Twelve interventions manipulated only dietary intake with a focus on bisphenol A (BPA) and phthalates, 8 studies intervened only on PCPs use and focused on a wider range of chemicals including BPA, phthalates, triclosan, parabens, and ultraviolet absorbers, while 6 studies intervened on both diet and PCPs and focused on phthalates, parabens, and BPA and its alternatives. No studies assessed glycol ethers. All but five studies reported results in the expected direction, with interventions removing potential sources of exposures lowering EDC concentrations and interventions providing exposures increasing EDC concentrations. Short interventions lasting a few days were successful. Barriers to intervention success included participant compliance and unintentional contamination of products.

**Summary:**

The identified interventions were generally successful but illustrated the influence of participant motivation, compliance, ease of intervention adherence, and the difficulty of fully removing exposures due their ubiquity and the difficulties of identifying “safer” replacement products. Policy which reduces or removes EDC in manufacturing and processing across multiple sectors, rather than individual behavior change, may have the greatest impact on population exposure.

**Supplementary Information:**

The online version contains supplementary material available at 10.1007/s40572-023-00394-8.

## Introduction

A growing body of evidence suggests that exposures to endocrine-disrupting compounds (EDC) such as phthalates, synthetic phenols, and glycol ethers have implications for human health including the potential to disrupt common endocrine pathways such as the thyroid, estrogen, and androgen pathways [1–5]. There is increasing concern among public health and governmental organizations over the risks from exposures to these compounds, particularly exposures occurring in vulnerable periods such as early life [6].

These compounds are commonly found in everyday life from industrial uses to consumer products such as personal care products (PCPs) and food contact materials where they are used as plasticizers, fixatives, solvents, antibacterial agents, and preservatives [4, 7]. The prevalent use of these chemicals in everyday products means that the majority of the population is habitually exposed while going about their daily lives.

A survey among US adults found that, on average, women used 12 products and men used 6 products daily; among French adults, women on average used 16 products, men 8 products, and parents used 6 products on their children under 3 years of age [8, 9]. This is of concern as PCPs use has been associated with exposures to phenols, phthalates, and glycol ethers [10–23]. Similarly, dietary consumption results in potential exposure to several phenols and phthalates due to their use in food processing or food contact materials, such as the use of parabens as preservatives and antifungal agents [24–26].

While European Union (EU) legislation (Regulation (EC) No 1223/2009; (EC) No 2023/2006; (EC) 1907/2006; (EC) No 1935/2004) for ingredients found in cosmetics and food contact materials exist to limit or ban their use in some products, there remain concerns that the current legislation is not adequate as, among others, it still permits the use of chemicals recognized as “Substances of Very High Concern” such as BPA and does not take into account recent knowledge on non-monotonic dose–response relationships, mixtures resulting from co- and aggregated use of products, and the effects of exposures occurring during sensitive periods of development [26–28]. In addition, despite these regulations and even while exposure levels have tended to decrease over time, recent studies have reported detection of these compounds at high frequencies in population cohorts [29–31]. Recent recognition of their potential impact includes the 2020 European Chemicals Strategy for Sustainability (COM/2020/667 final) with commitments to ensure all chemicals are used sustainably and safely and to reduce exposures to chemicals of concern by minimizing and substituting the use of harmful chemicals, particularly in consumer products [32, 33]. As part of those commitments, the European Commission has initiated reform processes of several pieces of chemicals legislation, such as the REACH regulation and a targeted review of the cosmetics regulation, which aim at achieving a higher level of protection against harmful chemicals; a review of the food contact materials legislation is also expected to begin shortly [34–36]. Furthermore, the European Food Safety Authority recently re-evaluated the risks of BPA and proposed a new tolerable daily intake threshold of 0.04 ng/kg body weight/day from the current TDI of 4 µg/kg body weight/day [37]. However, implementation of any regulatory measures or commitments may take time. It is therefore beneficial to identify interventions which could be carried out to reduce personal chemical burdens.

We undertook a scoping review to map and collate the available evidence on interventions in the general population which altered exposure to phthalates, glycol ethers, and common synthetic phenols including BPA, triclosan, parabens, and UV filters, present in the diet, food packaging, and in PCPs. This review aims to describe the range and nature of interventions in this area, identify gaps in study populations and designs, and summarize the effectiveness of different intervention types. Findings from this review will help to provide recommendations for reducing exposures to phthalates, glycol ethers, and phenols both for individuals as well as for health professionals providing advice and guidance.

## Methods

A scoping review was conducted to describe the characteristics, range, and extent of research evidence, characterize comparisons such as between interventions, identify existing gaps in the literature such as in populations studied, and rapidly summarize and disseminate findings [38–40]. We followed Arksey and O’Malley’s framework for conducting a scoping study: (1) identifying the research question; (2) identifying relevant studies; (3) study selection; (4) charting the data; (5) collating, summarizing, and reporting the results [40].

Our review was underpinned by the research question “What is known from the literature about studies that aim to intervene on exposures to phthalates, glycol ethers and synthetic phenols from personal care products and dietary intake?”. This question was informed by and complements the Advancing Tools for Human Early Lifecourse Exposome Research and Translation (ATHLETE) European project which has an aim of developing and implementing interventions to improve the chemical exposome occurring from PCPs, with a focus on exposures from phenols, phthalates, and glycol ethers (hereafter, “EDC of interest”) [41]. While synthetic phenols include BPA, triclosan, and parabens, they will be discussed as these sub-classes of phenols rather than as a group because they are used for different applications by industries and are commonly recognized as their sub-classes in the media, on consumer products, and by researchers. These compound families were chosen given their widespread exposure in general population and potential deleterious effects on human health [1–5]. The search was conducted by a research librarian using Healthcare Databases Advanced Search (HDAS) and the Medline, EMBASE, and CINAHL electronic databases and was conducted for personal care products and dietary intake separately (search terms are provided in Additional file 1). The searches were conducted in March 2021 and updated in October 2021 and March 2022 to include more recent publications. To characterize as large a range of studies as possible, we did not have exclusion criteria; studies were included if they described an intervention to alter exposures regardless of whether there was a separate health outcome of interest and the study assessed pre- and post-intervention exposure. We included studies that intentionally exposed participants to chemical-containing products to illustrate the impact of stopping use of these products; if biological concentrations increase following exposures then, accordingly, they should decrease if these products were removed.

### Personal Care Products

**Fig. 1:**
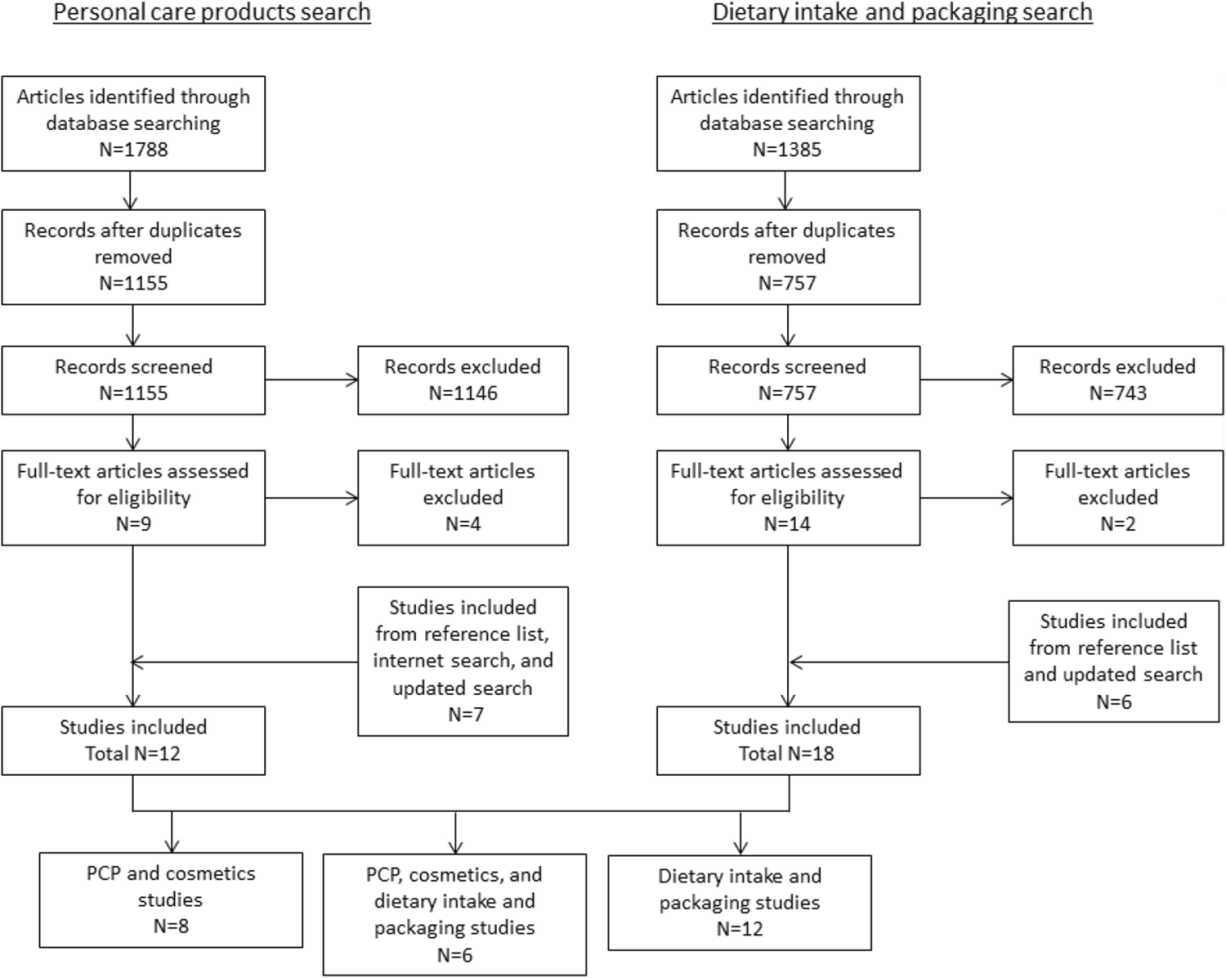
Flow diagram of the PCPs and dietary intake and packaging literature search

A total of 1788 articles were retrieved by the initial database search conducted in March 2021 and imported into the Rayyan Review platform (https://rayyan.qcri.org/) with 1 article identified from a web search (Fig. 1). A total of 1155 articles remained after de-duplication and titles and abstracts of the retained articles were screened and assessed for eligibility using two independent reviewers (TCY, FC); any disagreements were discussed and resolved between the two reviewers. *N* = 1146 articles were eliminated as not relevant because they did not focus on one of the EDC of interest or were not interventions, and full-text screening was conducted on the remaining nine articles. Four articles were excluded as the intervention was not on an EDC of interest and five articles were identified from the reference lists of the included studies and through an internet search. In the updated literature searches, titles and abstracts were screened by independent reviewers (October 2021: TCY, MW; March 2022: TCY, NJ) and two additional studies were identified and included. The relevant information from the 12 retained articles were recorded in a table and the data analyzed.

### Dietary Intake and Packaging

A total of 1385 articles were retrieved by the initial database search performed in March 2021 and imported into Microsoft Excel (Fig. 1). A total of 757 articles remained after de-duplication and titles and abstracts of the retained articles were screened and assessed for eligibility using two independent reviewers (NJ, CP); any disagreements were discussed and resolved between the two reviewers. *N* = 743 articles were eliminated as not relevant (i.e., not on EDC of interest or were not interventions) and full-text screening was conducted on the remaining 14 articles. Two articles were excluded. One had a small sample size (*N* = 5) and did not have a baseline sample since the first spot urine sample was collected 4 days after the removal of products made of plastics from the household [42] and the second was a weight loss intervention with no specific focus on sources of exposure to phenols, phthalates, or glycol ethers [43]. A total of three articles were identified from the reference lists of the included studies. In the updated search, titles and abstracts were screened by independent reviewers (October 2021: NJ, CP; March 2022: NJ, TCY) and three additional articles were identified and included. The relevant information from the 18 retained articles were recorded in a table and the data analyzed.

## Results

A total of 26 studies were included in this scoping review. Twelve studies were identified from the PCPs literature search and eighteen studies from the dietary intake and packaging literature search; six studies [44–47] were identified across both searches as the interventions targeted both PCPs and dietary intake. Studies are presented in separate tables for the PCPs literature (Table 1), dietary intake literature (Table 2), and studies targeting both PCPs and dietary intake and packaging (Table 3).

**Table 1:** Intervention studies targeting exposures from PCPs

Author (year)	Study population Origin	Chemicals	Pre-intervention		Intervention				Post-intervention			Results
			Concentration	No. and type of samples	About	Length	End of intervention concentration	No. and type of samples	Collection timepoint after intervention	Concentration	No. and type of samples	
Allmyr (2009)	*n* = 12 (*n* = 7 females; *n* = 5 males) Age (median [10–90th percentile]) years: 27 (23–47) Sweden	Triclosan	Median (range)^c^ ng/g 0.12 (0.009–0.81)	1; Blood plasma	Participants were instructed to brush their teeth for 3 min with a toothpaste containing 0.3% triclosan (Colgate Total) twice a day	14 days			1 day	Median (range)^c^ ng/g 55 (26–296)	1; Blood plasma	Plasma triclosan concentrations were higher after the 14-day treatment period
Huang (2021)	*n* = 10 females Age years: 22–26 China	Parabens	Mean (SD)^a^ ug/day Control period: Methylparaben: 76.42 (19.84) Ethylparaben: 0.64 (0.15) Propylparaben: 13.77 (3.60) Butylparaben: 0.40 (0.11) Benzylparaben: < 0.01 Metyl protocatechuate: 2.81 (0.67) Ethyl protocatachuate: 0.02 (0.01) 4-hydroxygenzic acid: 931.16 (560.30)	2 24 h; urine	Participants had 6 control days of standard chemical normal PCP use followed by 6 intervention days with low-chemical PCP products (facial cleanser, facial cream, toner), followed by 6 control days	18 days (12 control days, 6 treatment days)	Mean (SD)^a^ ug/day Intervention period: Methylparaben: 13.48 (3.31) Ethylparaben: 0.11 (0.04) Propylparaben: 3.88 (1.04) Butylparaben: 0.06 (0.01) Benzylparaben: < 0.01 Metyl protocatechuate: 0.51 (0.12) Ethyl protocatachuate: 0.03 (0.02) 4-hydroxygenzic acid: 690.37 (338.01)	1 24 h; urine				Urinary levels of parabens and their metabolites decreased during the intervention period compared to the control period
Harley (2016)	*n* = 100 females Age years: 14–18 US	Phthalates Parabens Triclosan Benzophenone-3	GM(SE)^b^ in µg/mL MEP: 78.2 (1.1) MnBP: 28.3 (1.1) MiBP: 15.2 (1.1) Methylparaben: 77.4 (1.2) Ethylparaben: 2.9 (1.2) Butylparaben: 0.8 (1.2) Propylparaben: 22.6 (1.3) Triclosan: 9.5 (1.3) Benzophenone-3: 173.8 (1.2)	1; urine	Participants were provided with education about chemicals in PCPs and makeup and provided with low-chemical PCPs (shampoo, conditioner, body wash, moisturizing lotion, bar of hand soap, liquid soap, roll-on deodorant, and a choice of 4 items from: liquid or powder foundation, eyeliner, mascara, lip balm/lipstick/lip gloss, sunscreen) and asked to refrain from using products other than those provided for 3 days. Toothpaste was provided only if participants used a particular brand which listed triclosan as an ingredient	3 days	GM(SE)^b^ in µg/mL MEP: 56.4 (1.1) MnBP: 25.2 (1.1) MiBP: 15.2 (2.3) Methylparaben: 43.2 (1.2) Ethylparaben: 4.2 (1.2) Butylparaben: 1.7 (1.2) Propylparaben: 12.3 (1.2) Triclosan: 6.1 (1.2) Benzophenone-3: 113.4 (1.2)	1; urine				Concentrations of phthalate metabolites, paraben, triclosan, and benzophenone-3 all decreased
Koch (2014)	*n* = 8 (*n* = 4 females; *n* = 4 males) Age years: 31–68 Belgium	Parabens Triclosan BPA Benzophenone-3	Mean (SD)^a^ µg/g Methylparaben: 168.2 (282.2) Ethylparaben: 35.5 (44.5) n-Propylparaben: 49.7 (161.1) Triclosan: 555 (501.1) Benzophenone-3: 62 (186.8) BPA: 2.5 (3.2)	Average of urine samples collected over the 4 control days	Participants’ usual PCPs were assessed (hair products, skin lotions, soaps, toothpastes, cosmetics, sunscreens, deodorants) and replacement products which did not contain any of the target analytes were identified. Participants used the replacement products on days 2 and 3 of the intervention period before using their usual products for the remainder of the days	6 days (4 control days; 2 intervention days)	Mean (SD)^a^ µg/g Methylparaben: 37.6 (51.6) Ethylparaben: 12.2 (14.7) n-Propylparaben: 3.3 (6.9) Triclosan: 294.7 (407.7) Benzophenone-3: 103.2 (279.3) BPA: 1.6 (1.1)	Average of urine samples collected over the intervention days				Paraben concentrations were lower during intervention days. No differences were observed for BPA and an increase was seen for benzophenone-3
Ley (2017)	*n* = 154 females (*n* = 78 exposed; *n* = 76 non-exposed) Age years: 18–42 US	Triclosan	median (IQR)^c^ pg/µL Exposed: 6.8 (1.4, 56.4) Non-exposed: 6.8 (2.7, 37.5)	1; urine	Participants in the exposed group were provided with commercially-available washing products which contained triclosan (dishwashing liquid, liquid and bar soaps, toothpaste). Non-exposed group participants were provided with the same products which did not contain triclosan	From baseline visit (~ 23 weeks gestation) to late pregnancy or post-delivery visit	median (IQR)^c^ pg/µL Exposed: 19 (3.1, 80.6) Non-exposed: 2.7 (0.3, 10.9)	Mean of urine samples: 1 at late pregnancy; 1 at post-delivery				Exposed group exhibited higher concentrations of triclosan compared to the non-exposed group
Janjua (2008a)	*n* = 26 males age [mean(sd)] years: 26 (4) Copenhagen, Denmark	Phthalates Parabens Phenols	Mean (SEM)^c^ µg/day: Control week: MEP: 565 (42) MBP: 116 (4.5) BP: 2.6 (0.5)	24-h urine samples combined over the control week	A control week was followed by a treatment week During the control week a body cream was applied daily to the whole body for 5 days. During the treatment week the cream contained 2% w/w of DEP, DBP, and BP was applied daily for 5 days	2 weeks (1 control week; 1 treatment week)	Mean (SEM)^c^ µg/day: Treatment week: MEP: 40,996 (1889) MBP: 11,754 (609) BP: 2,596 (136)	Average of 24-h urine samples over the treatment week				Following the treatment week, all 3 compounds were detectable in higher concentrations than during the control week
Janjua (2008b)	*n* = 32 (15 males; 17 females) Age [mean(sd)] years: male 26 (2) female = 65(12) Copenhagen, Denmark	Benzophenone-3	Median (range)^c^ ng/mL Females BP-3 Urine: < LOD Plasma: < LOD Males BP-3 Urine: < LOD Plasma: < LOD	1; urine, 1; blood plasma	On each day, participants had a sunscreen composed of 10% w/w of the chemical sunscreens benzophenone-3 applied to the whole body	4 days	Median (range)^c^ ng/mL Female BP-3 Urine: 17 (7–221) Plasma: 53 (0–802) Male BP-3 Urine: 71 (7–350) Plasma: 53 (11–226)	1; urine, 1; blood plasma				Concentration of benzophenone-3 was below the limit of detection prior to application of the sunscreen and was detectable at the end of the intervention period. Maximum plasma levels were achieved 4 h after application for the females and 3 h for the males
Poole (2016)	*n* = 16 (*n* = 11 female; *n* = 5 male) age (mean) years: 43 US	Triclosan	Median (IQR)^c^ pg/µL 24.4 (7.2, 59.4)	1; urine	Participants were randomized to either 4 control months where products without triclosan were to be used or to 4 treatment months where products containing triclosan were to be used. After 4 months in one condition, participants crossed over to the other condition for 4 months	8 months (4 months control; 4 months treatment)	Median^c^ pg/µL Control phase: 14.6 Treatment phase: 1548	5 urine samples per 4-month period, pooled				Urinary triclosan levels were higher in the treatment phase compared to the control phase

^a^Creatinine adjusted; ^b^SG adjusted, ^c^unstandardized

Abbreviations: 5-*oxo-MEHP/MEOHP*, mono(2-ethyl-5-oxo-hexyl) phthalate; 5-*OH-MEHP/MEHHP*, mono(2-ethyl-5-hydroxyhexyl) phthalate; *BPA*, bisphenol A; *BPF*, bisphenol F; *BPS*, bisphenol S; *CI*, confidence interval; *DEHP*, di(2-ethylhexyl) phthalate; *GM*, geometric mean; *GSD*, geometric standard deviation; *mL*, milliliter; *MCPP*, mono-(3-carboxy-propyl) phthalate; *MCNP*, mono-carboxy-isononyl phthalate; *MCOP*, mono-carboxy-isooctyl phthalate; *MEP*, monoethyl phthalate; *MECPP*, mono-(2-ethyl-5-carboxypentyl) phthalate; *MEHP*, mono-(2-ethyl-5-hydroxyhexyl) phthalate; *MBP*, monobutyl phthalate; *MBzP*, monobenzyl phthalate; *µg*, microgram; *MiBP*, monoisobutyl phthalate; *MMeP*, monomethyl phthalate; *ng*, nanogram; *nmol*, nanomole; *PCPs*, personal care products; *pg*, picogram; *SE*, standard error; *SG*, specific gravity; *µL*, microliter; *UV*, ultraviolet

**Table 2:** Intervention studies targeting exposures from dietary intake or food packaging

Author (year)	Study population Origin	Chemicals	Pre-intervention		Intervention				Post-intervention			Results
			Concentration	No. and type of samples	About	Length	End of intervention concentration	No. and type of samples	Collection timepoint after intervention	Concentration	No. and type of samples	
Bae 2015	*n* = 60 (*n* = 4 males; *n* = 56 females) Age (mean [SD]) years: 73.1 (4.2) Korea	BPA	N/A	N/A	Participants were asked to visit the study site 3 times with ≥ 1-week interval between visits. All participants were asked to fast for ≥ 8 h and visit the study site at 9 am. Two servings of soy milk were provided to the participants in 3 different combinations at each time: 2 cans (CC); 2 glass bottles (GG); or 1 can and 1 glass bottle (CG)	2 servings of soy milk (urine collected 2 h after consumption)	Mean (SD)^a^ µg/L CC: 20.65 (8.61) CG: 9.43 (5.01) GG: 1.25 (2.26)	1 (after each combination); urine				Consuming canned soy milk increased urinary BPA concentrations compared to consuming the same beverage in glass bottles
Barrett (2015)	*n* = 10 pregnant females Age (mean [SD]) years: 26.4 years (5.0) US	Phthalates	GM (SD)^b^ µg/L MEHP: 2.8 (3.2) MEOHP: 10.0 (1.9) MEHHP: 12.2 (2.3) MECCP: 24.1 (2.0) ∑DEHP: 171.8 (19.9) MBP: 19.4 (1.6) MEP: 72.3 (5.2) MiBP: 15.9 (1.5) MBzP: 10.0 (3.2) MCPP: 4.4 (3.6) MCNP: 3.7 (2.5) MCOP: 27.7 (3.9)	1; urine	Participants were provided with a diet consisting of mostly organic and fresh foods. All food was prepared and stored without the use of plastic utensils and containers	3 days	GM (SD)^b^ µg/L MEHP: 3.2 (2.0) MEOHP: 13.2 (1.8) MEHHP: 17.0 (2.1) MECCP: 30.6 (1.6) ∑DEHP: 219.6 (16.8) MBP: 24.6 (2.1) MEP: 125.9 (10.9) MiBP: 24.0 (2.0) MBzP: 15.7 (2.2) MCPP: 4.4 (2.1) MCNP: 5.2 (1.5) MCOP: 29.4 (2.2)	1; urine	3 days after the end of intervention	GM (SD)^b^ µg/L MEHP: 3.6 (2.1) MEOHP: 12.7 (2.0) MEHHP: 18.4 (2.1) MECCP: 33.9 (2.2) ∑DEHP: 233.3 (20.6) MBP: 27.5 (1.7) MEP: 108.4 (4.3) MiBP: 23.7 (1.5) MBzP: 14.2 (2.4) MCPP: 7.0 (3.2) MCNP: 5.2 (2.6) MCOP: 41.3 (4.5)	1; urine	All urinary concentrations increased from pre- to post-intervention but only statistically significantly for MiBP
Carwile (2009)	*n* = 77 (*n* = 41 males; *n* = 36 females) Age years: 18–23 UK	BPA Negative controls: Parabens Triclosan Benzophenone-3	GM (95% CI)^a^ µg/L after washout BPA: 1.2 (1.0–1.4) Methylparaben: 51.3 (37.3–70.7) Propylparaben: 8.4 (5.4–12.9) Triclosan: 15.5 (9.5–25.3) Benzophenone-3: 46.1 (30.6–69.5)	1; urine	The study began with a 7-day washout phase designed to minimize exposure to BPA by limiting the consumption of cold beverages to those contained in stainless steel bottles. Participants were then given polycarbonate bottles and advised them to start drinking cold beverages from it for the duration of the intervention period	7 days	GM (95% CI)^a^ µg/L BPA: 2.0 (1.7–2.4) Methylparaben: 48.4 (36.2–64.8) Propylparaben: 8.8 (5.8–13.1) Triclosan: 17.3 (10.7–28.1) Benzophenone-3: 66.8 (42.3–105.5)	1; urine				The use of polycarbonate bottles for cold beverage drinking increased urinary concentrations of BPA and benzophenone-3
Carwille (2011)	*n* = 75 (*n* = 24 males; *n* = 51 females) Age (median [range]) years: 27 (22–56) US	BPA	N/A	N/A	For the first 5-day period, one group consumed a 12-oz serving of fresh soup (prepared without canned ingredients) daily; the other group consumed a 12- ounce serving of canned soup. After a 2-day wash-out, treatment assignments were reversed. There were no restrictions on the consumption of other foods	5 days, twice	Mean (95% CI)^b^ µg/L After canned soup consumption: 20.8 (17.9–24.1) After fresh soup consumption: 1.1 (0.8–1.4)	2 (day 4 and 5 of each period); Urine				Consumption of canned soup increased urinary BPA concentrations
Galloway (2017)	*n* = 94 (*n* = 41 males; *n* = 63 females) Age: 17–19 years UK	BPA	Mean (SD)^a^ ng/mL BPA: 1.58 (1.64)	1; urine	The research team and participants co-designed a set of recommendations on processed and packaged food to be avoided during the intervention	7 days	Mean (SD)^a^ ng/mL BPA: 3.13 (7.36)	1; urine				BPA concentrations did not decrease, even with good compliance
Ji (2010)	*n* = 25 participants (*n* = 16 males; *n* = 9 females) Age (mean [SD]) years: 37.6 (13.3) Korea	Phthalates	Median^a^ ng/mg Male MEP: 44.8 MnBP: 74.8 MiBP: 18.6 MEHP: 10.6 5-oxo-MEHP: 21.9 5-OH-MEHP: 42.4 Female MEP: 30.8 MnBP: 136.7 MiBP: 31.6 MEHP: 13.9 5-oxo-MEHP: 35.3 5-OH-MEHP: 43.1	1; urine	Participants followed a strict Buddhist vegetarian diet and followed the daily routines of Buddhist monks	5 days	Median^a^ ng/mg Male MEP: 17.3 MnBP: 27.7 MiBP: 7.5 MEHP: 9.2 5-oxo-MEHP: 15.6 5-OH-MEHP: 18.7 Female MEP: 3.2 MnBP: 36.6 MiBP: 9.2 MEHP: 10.6 5-oxo-MEHP: 18.4 5-OH-MEHP: 23.0	1; urine				Urinary concentrations of MEP, MnBP, MiBP, 5-oxo-MEHP, and 5-OH-MEHP significantly during the intervention
Jo (2020)	*n* = 25 participants (*n* = 16 males; *n* = 9 females) Age years: 13–64 Korea	Parabens Benzophenones	GM^a^ µg/g Whole population Methylparaben: 52.7 Ethylparaben: 8.72 Propylparaben: 6.83 Butylparaben: 2.93 Benzophenone-1: 2.56 Benzophenone-3: 4.67 Male Methylparaben: 30.4 Ethylparaben: 8.73 Propylparaben: 2.54 Butylparaben: 2.14 Benzophenone-1: 1.54 Benzophenone-3: 3.2 Female Methylparaben: 140 Ethylparaben: 8.7 Propylparaben: 39.7 Butylparaben: 5.1 Benzophenone-1: 6.29 Benzophenone-3: 9.14	1; urine	Parcticipants followed a strict Buddhist vegetarian diet and followed the daily routines of Buddhist monks	5 days	GM^a^ µg/g Whole population Methylparaben: 54.5 Ethylparaben: 52.6 Propylparaben: 10.5 Butylparaben: 1.03 Benzophenone-1: 2.87 Benzophenone-3: 3.23 Male Methylparaben: 25.9 Ethylparaben: 58.6 Propylparaben: 3.59 Butylparaben: 0.49 Benzophenone-1: 1.82 Benzophenone-3: 2.98 Female Methylparaben: 205 Ethylparaben: 43.5 Propylparaben: 72.3 Butylparaben: 3.80 Benzophenone-1: 6.44 Benzophenone-3: 3.74	1; urine				No significant changes in urinary concentrations were observed among the whole population and among females. Among males, ethylparaben increased while butylparaben decreased
Kim (2020)	*n* = 93 (*n* = 37 mothers; *n* = 56 children recruited from 37 families) Age: children: 4–12 years mothers: 30–50 years Korea	BPA, BPS	GM (GSD)^b^ ng/mL Children BPA: 1.29 (0.03) BPS: 0.20 (0.26) Mothers BPA: 0.87 (0.02) BPS: 0.19 (0.35)	2; urine	Participants were asked to refrain from, instant foods, foods in cans and plastic containers, and delivery food	3 days	GM (GSD)^b^ ng/mL Children BPA: 0.71 (0.05) BPS: 0.12 (0.41) Mothers BPA: 0.42 (0.15) BPS: 0.07 (0.62)	2 pooled; urine				BPA decreased in both mothers and children BPS decreased in mothers
Park (2021)	*n* = 30 female Age (mean[SD]) years: 22.1 (1.5) Korea	BPA	Median (min–max)^a^ µg/g BPA: 0.99 (0.22–3.99)	1; urine	The intervention involved dietary modifications and targeted reduction of processed/fast foods and included: (1) education about EDCs, how to avoid them, and their impact on reproductive health; (2) follow-up monitoring for 4 weeks; (3) peer support via social network	4 months (1-month intervention; 3 months follow-up at timing of menstrual cycle)			3 months at each menstrual cycle	Median (min–max)^a^ µg/g BPA: Month 1: 0.41 (0.06–1.42) Month 2: 0.45 (0.07–1.42) Month 3: 0.72 (0.08–2.45)	3; urine	BPA decreased in the 1st and 2nd menstrual cycles compared to baseline but not at the 3rd menstrual cycle
Peng (2019)	*n* = 20 (*n* = 10 males; *n* = 10 females) Age years: 21–32 Taiwan	BPA	Mean (SE)^a^ µg/g Day 2: canned food BPA: 7.4 (3.6) Day 2: fresh food BPA: 5.4 (2.4) Day 4: canned food BPA: 3.9 (2.0) Day 4: fresh food BPA: 4.2 (1.9)	1; urine	On the first day of the intervention, participants were asked to avoid drinking coffee, canned food, and plastic-packaged food, and to not eat after 10 PM. On the second day, participants were randomly assigned in one of the two groups (breakfast made from canned food or fresh food). The third day was a wash-out and on the fourth day the participants received the alternate group (breakfast made from canned food or fresh food)	4 days (1 day wash-out, 1 day one condition, 1 day wash-out, 1 day alternate condition)	Mean (SE)^a^ µg/g Day 2: canned food BPA Breakfast + 2 h: 10.6 ± 1.5 Breakfast + 4 h: 20.4 ± 8.9 Breakfast + 6 h: 12.5 ± 2.9 Day 2: fresh food BPA Breakfast + 2 h: 7.2 ± 2.5 Breakfast + 4 h: 7.0 ± 1.9 Breakfast + 6 h: 6.7 ± 1.5 Day 4: canned food BPA Breakfast + 2 h: 8.4 ± 3.1 Breakfast + 4 h: 13.3 ± 6.3 Breakfast + 6 h: 6.6 ± 1.9 Day 4: fresh food BPA Breakfast + 2 h: 3.9 ± 1.3 Breakfast + 4 h: 2.5 ± 0.8 Breakfast + 6 h: 4.6 ± 2.3	3 (2 h, 4 h, and 6 h after breakfast)				Canned food consumption increased urinary BPA concentrations, which peaked 4 h after ingestion. Fresh food consumption did not change BPA concentrations
Rudel (2011)	*n* = 20 from 5 families (*n* = 9 males; *n* = 11 females; *n* = 10 adults; *n* = 10 children) Age (median) years: adults: 40.5 children: 7 US	BPA Phthalates	GM^c^ ng/mL BPA: 3.7 MEHP: 7.1 MEOHP: 27 MEHHP: 57 MEP: 41 MBP: 43 MBzP: 12 MMEP: 12	2; urine	Participants consumed food provided by the research team made with fresh and organic fruits, vegetables, grains, and meats. Participants received stainless steel water bottles and lunch containers to avoid other common sources of BPA and phthalates. If they had to depart from the provided foods, participants were advised to eat fresh foods, such as fruits, vegetables, eggs, peanut butter, and jelly from glass jars, and milk and orange juice from glass containers or low-density polyethylene plastic if glass was not available	3 days	GM^c^ ng/mL BPA: 1.2 MEHP: 3.4 MEOHP: 12 MEHHP: 25 ME:P 50 MBP: 32 MBzP: 10 MMEP: 12	2; urine	Day 2 and 3 after the end of intervention	GM^c^ ng/mL BPA: 3.8 MEHP: 4.1 MEOHP: 14 MEHHP: 31 MEP: 53 MBUP: 35 MBzP: 11 MMEP: 9.3	2; urine	BPA, MEHP, MEOHP, and MEHHP decreased during the intervention while BPA increased post-intervention
Sathyanarayana (2013)	*n* = 40 (*n* = 21 intervention; *n* = 19 control; *n* = 11 male; *n* = 10 female; *n* = 10 adults; *n* = 11 children) Age (mean) years: adults: N/A children: intervention: 6 control: 5 US	BPA Phthalates	GM(95%CI)^c^ µg/L Intervention BPA: 0.8 (0.6, 1.0) MEP: 18.0 (10.6, 30.6) MBP: 14.7 (8.8, 24.5) MBzP: 6.9 (3.7, 13.1) MEHP: 4.8 (2.4, 9.6) MEHHP: 31.8 (16.9, 59.8) MEOHP: 17.8 (9.5, 33.2) MCEPP: 27.5 (15.5, 49.0) Control BPA: 1.4 (0.9, 2.2) MEP: 16.9 (11.6, 24.6) MBP: 14.9 (10.1, 21.9) MBzP: 8.6 (5.0, 14.9) MEHP: 3.9 (2.8, 5.3) MEHHP: 22.0 (16.3, 29.8) MEOHP: 12.3 (9.0, 16.7) MCEPP: 24.4 (17.5, 34.0)	1; urine	A two-arm, randomized study that compares the efficacy of complete dietary replacement with fresh and organic, catered foods prepared without plastics (intervention) versus education using handouts describing best practice recommendations to reduce exposures to phthalates and BPA (control)	5 days	GM(95%CI)c µg/L Intervention BPA: 1.6 (1.1, 2.3) MEP: 52.0 (29.2, 92.6) MBP: 23.0 (15.9, 33.2) MBzP: 11.5 (6.3, 21.2) MEHP: 84.8 (49.9, 144.1) MEHHP: 834.2 (528.1, 1317.7) MEOHP: 834.2 (528.1, 1317.7 MCEPP: 706.7 (434.8, 1148.7) Control BPA: 1.4 (1.0, 2.1) MEP: 28.0 (15.5, 50.4) MBP: 17.0 (12.6, 23.1) MBzP: 10.4 (6.8, 15.8) MEHP: 4.1 (2.8, 5.9) MEHHP: 21.8 (15.9, 29.9) MEOHP: 12.6 (9.7, 16.5) MCEPP: 23.1 (16.9, 31.5)	1; urine	6 days after the end of intervention	GM(95%CI)c µg/L Intervention BPA: 2.0 (1.1, 3.5) MEP: 41.8 (22.9, 76.5) MBP: 16.1 (9.5, 27.3) MBzP: 10.4 (5.0, 21.8) MEHP: 2.8 (1.4, 5.7) MEHHP: 21.2 (11.7, 38.6) MEOHP: 12.5 (7.1, 21.9) MCEPP: 19.9 (12.1, 33.0) Control BPA: 1.5 (1.0, 2.4) MEP: 21.4 (14.2, 32.3) MBP: 20.0 (14.4, 27.9) MBzP: 10.8 (6.8, 15.8) MEHP: 4.2 (3.2, 5.6) MEHHP: 23.3 (16.2, 32.3) MEOHP: 14.0 (9.9, 19.8) MCEPP: 27.9 (18.5, 42.1)	1; urine	Urinary concentrations of BPA, MEP, MBP, MEHP, MEHHP, MEOHP, MECPP increased from baseline to intervention which was an artefact arising from contamination of the food provided to participants. MEHP, MEHHP, MEOHP, MECPP subsequently decreased post-intervention. No significant changes were observed among control group participants

^a^Creatinine adjusted; ^b^SG adjusted, ^c^unstandardized

Abbreviations: 5-*oxo-MEHP/MEOHP*, mono(2-ethyl-5-oxo-hexyl) phthalate; 5-*OH-MEHP/MEHHP*, mono(2-ethyl-5-hydroxyhexyl) phthalate; *BPA*, bisphenol A; *BPF*, bisphenol F; *BPS*, bisphenol S; *CI*, confidence interval; *DEHP*, di(2-ethylhexyl) phthalate; *GM*, geometric mean; *GSD*, geometric standard deviation; *mL*, milliliter; *MCPP*, mono-(3-carboxy-propyl) phthalate; *MCNP*, mono-carboxy-isononyl phthalate; *MCOP*, mono-carboxy-isooctyl phthalate; *MEP*, monoethyl phthalate; *MECPP*, mono-(2-ethyl-5-carboxypentyl) phthalate; *MEHP*, mono-(2-ethyl-5-hydroxyhexyl) phthalate; *MBP*, monobutyl phthalate; *MBzP*, monobenzyl phthalate; *µg*, microgram; *MiBP*, monoisobutyl phthalate; *MMeP*, monomethyl phthalate; *ng*, nanogram; *nmol*, nanomole; *PCPs*, personal care products; *pg*, picogram; *SE*, standard error; *SG*, specific gravity; *µL*, microliter; *UV*, ultraviolet; *US*, United States; *UK*, United Kingdom; *LOD*, limit of detection

**Table 3:** Intervention studies targeting exposures from PCPs and dietary intake or food packaging

Author (year)	Study population Origin	Chemicals	Pre-intervention visit		Intervention				Post-intervention visit			Results
			Concentration	No. and type of samples	About	Length	End of intervention concentration	No. and type of samples	Collection timepoint after intervention	Concentration	No. and type of samples	
Chen (2015)	*n* = 30 females Age years (median): 4–13 (9.7) Taiwan	Phthalates	Median (range)^a^ µg/g MMP: 10. (2.75, 29.7) MEP: 39.2 (9.08, 650) MBP: 117 (55.5, 482) MBzP: 6.62 (1.50, 58.2) MEHP: 11.4 (3.43, 38.3) MEOHP: 41.2 (19.8, 207) MEHHP: 82.1 (32.9, 398) MECPP: 83.0 (34.7, 320) ΣDEHP: 0.71 (0.34, 3.21)	1; urine	Participants were requested to change or refrain from certain behaviors including refraining from cosmetics and PCPs, use of plastic containers and nutrition supplements and medication, microwaved food, consuming food in plastic bags or wrapped in plastic, certain building materials, and to wash their hands prior to consuming food	7 days			1 day	Median (range)^a^ µg/g MMP: 9.42 (2.97, 26.5) MEP: 33.0 (4.66, 302) MBP: 98.2 (36.3, 505) MBzP: 5.66 (1.25, 68.1) MEHP: 8.95 (3.42, 208) MEOHP: 33.0 (15.5, 735) MEHHP: 68.3 (29.1, 1848) MECPP: 67.7 (30.1, 1433) ΣDEHP: 0.60 (0.27, 14.2)	1; urine	Phthalate concentrations pre-intervention were slightly higher than the post-intervention sample. Only urinary creatinine-adjusted MBzP was lower post-intervention Analyses restricted to those who were compliant found significantly lower concentrations for all phthalate metabolites post-intervention compared to pre-intervention
El Ouazzani (2022)	*n* = 230 (*n* = 152 intervention; *n* = 78 control) pregnant women Age (mean) years: 33 France	Parabens BPA	Mean^c^ ng/mL Control Methylparaben: 15.8 BPA: values not provided Intervention Methylparaben: 13.6 BPA: values not provided	1; urine	A three-arm randomized controlled trial with control group receiving informational leaflets on EDCs, an intervention arm receiving leaflets and workshops on indoor air quality, nutrition, and PCPs conducted in a neutral location (meeting room), and an intervention arm receiving leaflets and workshops on indoor air quality, nutrition, and PCPs conducted in a contextualized location (apartment). Intervention arms combined in analyses. Study only reported values for methylparaben and BPA	Workshops were carried out between the 2nd and 3rd trimesters of pregnancy	Mean^c^ ng/mL Control Methylparaben: 7 BPA: values not provided Intervention Methylparaben: 14.8 BPA: values not provided	1; urine	1-year post-birth	Mean^c^ ng/mL Control Methylparaben (urine): 3 Methylparaben (colostrum): 0.28 BPA (colostrum): 1.16 Intervention Methylparaben (urine): 0.4 Methylparaben (colostrum): 0.21 BPA (colostrum): 1	1; urine 1; colostrum	No difference in BPA or paraben concentrations between the control and intervention arms at each of the collection periods (2nd trimester, 3rd trimester, 1 year postpartum)
Hagobian (2016)	*n* = 24 females Age (mean[SD]) years: control = 21.4 (1.5) intervention = 20.5 (1.5) US	BPA	GM (95% CI)^a^ µg/g Control: 1.06 (0.67, 1.66) Intervention: 1.59 (1.05, 2.41)	1; urine	Participants were randomly assigned to the control or intervention group Control group: participants received weekly email newsletter providing information about BPA, physical activity, and healthy eating Intervention group: Participants received the same materials as the control group and an additional behavioral intervention designed to reduce BPA exposure including weekly face-to-face meetings with a counsellor. Sessions included discussions on the health effects of BPA exposure, how to avoid exposure in diet and PCPs. Participants were additionally provided with BPA-free glass food storage containers, water bottles, and PCPs and cosmetics that were packaged in BPA-free packaging	3 weeks	GM (95% CI)^a^ µg/g Control: 1.37 (0.87, 2.15) Intervention: 0.88 (0.58, 1.34)					BPA concentrations in the intervention group decreased over the intervention period while concentrations increased in the control group
Habogian (2021)	*n* = 30 females with obesity (*n* = 15 control; *n* = 15 intervention) Age (mean [SD]) years: control = 21.5 (3.1) intervention = 21.5 (3.3) US	BPA BPS BPF	GM (95% CI)^a^ µg/g Control: BPA: 0.19 (− 0.21, 1.27) BPS: 0.73 (0.58, 2.34) BPF: 0.13 (− 4.55, 3.98) Intervention: BPA: 0.32 (0.25, 1.55) BPS: 2.31 (2.03, 3.81) BPF: 0.79 (1.73, 10.25)	1; urine	Same study design as Habogian (2016) but among participants with obesity	3 weeks	GM (95% CI)^a^ µg/g Control: BPA: 0.24 (0.08, 1.03) BPS: 0.64 (0.86, 2.06) BPF: 0.12 (− 0.14, 2.41) Intervention: BPA: 0.32 (0.19, 1.15) BPS: 1.04 (0.69, 1.89) BPF: 0.24 (− 0.54, 2.01)	1; urine				BPS concentrations decreased in the intervention group compared to the control group over the intervention period. No changes were observed with urinary concentrations of BPA or BPF
Kim (2021)	*n* = 51 females (*n* = 25 control; *n* = 26 intervention) Age (mean [SD]) years: control = 35.1 (2.9) intervention = 35.8 (3.9) Korea	Phthalates	GM (95%CI)^a^ in ug/g Control group: MEHP: 2.76 (1.26, 14.51) MEOHP: 4.20 (1.67, 33.40) MEHHP: 8.28 (1.83, 58.59) BPA: 0.80 (0.07, 5.90) Triclosan: 0.12 (0.04, 6.44) Methylparaben: 17.76 (3.64, 953.06) Ethylparaben: 49.16 (2.07, 2401.07) Propylparaben: 0.60 (0.08, 110.23) Intervention group: MEHP: 2.65 (1.07–13.09) MEOHP: 4.28 (1.70, 30.92) MEHHP: 8.60 (2.87, 43.10) BPA: 0.87 (0.53, 8.92) Triclosan: 0.13 (0.01, 6.39) Methylparaben: 16.76 (3.86, 639.94) Ethylparaben: 51.38 (1.17, 2742.91) Propylparaben: 0.70 (0.20, 87.19)	1; urine	Participants were randomized to the control or intervention group Control group: Participants were sent written information about EDCs by mail including their identification, health effects, and methods of reducing exposure Intervention group: Participants were encouraged to access a web-based program at least 3 times a week. The program consisted of five components: (1) educational video detailing the health effects of EDCs and ways of reducing exposure; (2) a game to identify items containing EDCs in the home; (3) search for local facilities such as gyms to faciliate exercise to release EDCs; (4) resources on how to avoid EDCs such as increasing intake of organic food, washing hands frequently, using glass or stainless steel cookware, and reducing intake foods with high fat content, strongly-scented PCPs and cosmetics, new furniture and cars	1 month	GM (95%CI)^a^ in ug/g Control group: MEHP: 2.67 (1.87, 13.84) MEOHP: 3.99 (1.01, 33.61) MEHHP: 8.22 (1.13, 68.88) BPA: 0.64 (0.07, 5.12) Triclosan: 0.15 (0.03, 10.20) Methylparaben: 20.09 (3.98, 771.12) Ethylparaben: 42 (11.07, 813.91) Propylparaben: 0.64 (0.04, 113.62) Intervention group: MEHP: 2.20 (1.02, 7.31) MEOHP: 3.33 (1.07, 13.96) MEHHP: 7.63 (3.14, 51.02) BPA: 0.40 (0.23, 4.11) Triclosan: 0.12 (0.06, 10.33) Methylparaben: 11 (3.55, 111.62) Ethylparaben: 33 (14.67, 566.31) Propylparaben: 0.31 (0.17, 43.15)	1; urine				Concentrations of MEHP, MEOHP, BPA, methyl-, ethyl-, and propyl-parabens decreased. No changes in triclosan concentrations were observed
Wu (2021)	*n* = 35 pregnant women Age years: 26–34 China	Phthalates	Mean (SE)^a^ (µg/g) MMP: 36.3 (27.2) MEP: 41.8 (22.7) MiBP: 24.8 (18.8) MnBP: 83.5 (43.1) MBzP: 2.41 (2.71) MoP: 1.00 (1.69) MEHP: 5.78 (6.96) MECPP: 17.9 (12.0) MEHHP: 88.0 (57.8) MEOHP: 85.0 (57.4) ∑mLPAE: 189 (58.6) ∑mDEHP: 196 (80.3)	1; urine	Participants were provided with written recommendations at three time periods (baseline; 2nd trimester — 4th month; 3rd trimester — 7th month) on: (1) diet, including restricting consumption of canned and microwaved food and increase consumption of healthy food, including organic foods; (2) lifestyle, including restricting PCPs and food stored in plastic containers; (3) environment, including increasing physical activity and reducing exposure to second-hand smoke. Urines were collected monthly; baseline, end of intervention (8th month), and follow-up (9th month) are reported	7 months	Mean (SE)^a^ (µg/g) MMP: 22.7 (15.9) MEP: 26.6 (16.8) MiBP: 14.8 (10.6) MnBP: 39.4 (21.8) MBzP: 2.65 (2.58) MoP: 1.36 (1.24) MEHP: 5.34 (6.14) MECPP: 8.13 (5.91) MEHHP: 48.8 (36.4) MEOHP: 53.9 (44.6) ∑mLPAE: 108 (33.0) ∑mDEHP: 116 (58.0)	1; urine	1 month after the end of the intervention	Mean (SE)^a^ (µg/g) MMP: 19.2 (13.5) MEP: 25.6 (16.8) MiBP: 12.5 (8.97) MnBP: 39.8 (24.1) MBzP: 2.69 (2.60) MoP: 1.29 (1.23) MEHP: 4.65 (6.07) MECPP: 7.07 (5.14) MEHHP: 49.1 (32.9) MEOHP: 51.0 (33.2) ∑mLPAE: 101 (34.7) ∑mDEHP: 112 (48.0)	1; urine	All concentrations decreased by the 9th month compared to baseline except for MBzP, which remained stable, and MEHP, which decreased after the 7th month

^a^Creatinine adjusted; ^b^SG adjusted, ^c^unstandardized

Abbreviations: 5-*oxo-MEHP/MEOHP*, mono(2-ethyl-5-oxo-hexyl) phthalate; 5-*OH-MEHP/MEHHP*, mono(2-ethyl-5-hydroxyhexyl) phthalate; *BPA*, bisphenol A; *BPF*, bisphenol F; *BPS*, bisphenol S; *CI*, confidence interval; *DEHP*, di(2-ethylhexyl) phthalate; *GM*, geometric mean; *GSD*, geometric standard deviation; *mL*, milliliter; *MCPP*, mono-(3-carboxy-propyl) phthalate; *MCNP*, mono-carboxy-isononyl phthalate; *MCOP*, mono-carboxy-isooctyl phthalate; *MEP*, monoethyl phthalate; *MECPP*, mono-(2-ethyl-5-carboxypentyl) phthalate; *MEHP*, mono-(2-ethyl-5-hydroxyhexyl) phthalate; *MBP*, monobutyl phthalate; *MBzP*, monobenzyl phthalate; *µg*, microgram; *MiBP*, monoisobutyl phthalate; *MMeP*, monomethyl phthalate; *ng*, nanogram; *nmol*, nanomole; *PCPs*, personal care products; *pg*, picogram; *SE*, standard error; *SG*, specific gravity; *µL*, microliter; *UV*, ultraviolet

Eight studies measured only bisphenols [44, 45, 48–53], four measured only phthalates [46, 54–56], two measured only triclosan [57, 58], and one measured only parabens [59], while ten assessed a mix of compounds [47, 50, 60–68]. None assessed glycol ethers. There was a range of study designs. Nine study designs involved providing participants with products containing EDC of interest [49, 52, 53, 57, 58, 61–64], six aimed to change participant behavior through only providing information on how to avoid phenols and phthalates [47, 48, 51, 56, 67, 68], while others involved dietary changes or actively removing and/or replacing products containing the EDC of interest with products without the EDC of interest, or a combination of both [44–46, 50, 55, 59, 60, 64–66, 69]. Five studies included control groups in addition to the intervention group [44, 45, 58, 66, 68], while five studies were cross-over trial designs [49, 52, 53, 63, 69]. Ten studies were conducted in non-Western countries [46, 47, 49–51, 56, 59, 67] with the remaining 16 conducted in Western countries, of which 10 were carried out in the US [44, 45, 52, 54, 58, 60, 63–66, 68]. The studies included population groups ranging from young children [46, 65, 67] to the elderly [53]. Some studies targeted specific groups such as pregnant women [54, 56, 58, 68] or women with overweight or obesity [45], while others included families [47, 65–67]. The majority of studies collected urine samples to examine biomarker concentrations; one study collected both blood and urine samples [61] while a second focusing on triclosan measured only in blood samples [57]. While blood is not the preferred matrix for chemicals with short half-lives, as those studied in this review, this study was kept since the frequency of detection was quite good for triclosan.

### Interventions on PCPs

Eight intervention studies which only altered chemical exposures from PCPs targeted BPA [60], phthalates [60, 62], parabens [62, 69], triclosan [57, 58, 69], and ultraviolet (UV) filters such as benzophenone-3 [61, 69] which are commonly found in everyday PCPs such as shampoo, body wash, deodorant, cosmetics, toothpaste, and sunscreens (Table 1). All studies but one [59] were conducted in Western countries. Three of these studies focused on decreasing exposure through removal or replacement of PCPs [59, 60, 69] while the remaining five aimed at increasing exposure by providing specific PCPs such as sunscreen and toothpaste which contained the chemicals of interest [57, 58, 61–63]. Studies which provide PCPs containing the chemicals of interest, and which report an increase in biomarker concentrations following their use, illustrate that removing these products can have a measurable effect on biomarkers of the EDC of interest. There were no studies utilizing an education-only approach. Interventions lasted from 2 days [62] to the length of pregnancy [58] and all studies reported the majority of their results in the expected direction; studies removing exposures found reductions in biomarker concentrations and studies providing exposures found increases in biomarker concentrations of the EDC of interest.

#### Interventions Aiming to Reduce Exposures

Three studies removed or replaced PCPs to understand their impact on urinary concentrations of the EDC of interest [59, 60, 69] (Table 1).

In a US study among 100 Latina girls aged 14–18 years, researchers provided a selection of replacement PCPs and cosmetics (including shampoo, conditioner, body wash, hand soap, deodorant, moisturizing lotion, and a choice of cosmetics including foundation, sunscreen, eye and lip make-up) which were chosen by using label information and online consumer databases to identify products free from triclosan, phthalates, parabens, and the UV absorber benzophenone-3 [60]. Participants had their usual PCPs substituted with these replacement products during the 3-day intervention and were allowed to choose four replacement cosmetics items to increase compliance to the change in beauty products during the intervention phase. The authors found reductions from pre- to post-intervention in urinary monoethyl phthalate (MEP), methylparaben, propylparaben, triclosan, and benzophenone-3 while concentrations increased over the intervention period for butylparaben. These reductions were larger among participants who used products known to contain triclosan and benzophenone-3 within 48 h of the pre-intervention visit. No changes from pre- to post-intervention were observed for monobutyl phthalate (MBP), monoisobutyl phthalate (MiBP), or ethyl paraben.

A cross-over study among Chinese women (age: 22–26 years) examined changes in paraben concentrations where women followed 6 days of typical PCPs use with a 6-day intervention of low-chemical PCPs product use (including facial cleaner, cream, and toner) followed by 6 days of typical PCPs use [59]. The authors found urinary levels of parabens and their metabolites decreased during the intervention period compared to combined 12 days (6 days prior to the intervention period and 6 days following the intervention period) where women were using their typical PCPs.

Only one study replacing PCPs included males [69]. This study conducted in Belgium recruited 8 participants (4 females, 4 males; 31–68 years old) for a 2-day intervention period. Using the ingredient lists, researchers replaced the usual PCPs used by the participants (including shampoo, hair conditioner, body and hand soap and gels, toothpaste, sunscreen, deodorant, make-up, and anti-bacterial products such as hand sanitizers) for products which did not contain the target analytes (parabens, benzophenones, triclosan, triclocarban, BPA). Compared to the average urinary concentrations while using their usual PCPs, urinary concentrations were reduced for methylparaben, ethylparaben, propylparaben, and triclosan but not benzophenone-3 or BPA.

#### Interventions Aiming to Increase Exposures

Five studies provided participants with products that contained the EDC of interest to understand absorption, metabolism, and excretion [57, 58, 61–63] (Table 1). Three studies monitored urinary biomarkers of exposure as well as potential biomarkers of effects including thyroid hormone concentration and function [57, 58] and the microbiome [63].

In a single-blinded Danish study of 26 men (mean age: 26 years) with normal weight, a control week was followed by a 4-day intervention where participants applied a body cream with added diethyl phthalate, dibutyl phthalate, and butyl paraben [62]. Urinary concentrations of the corresponding phthalates metabolites and the un-metabolized butyl paraben increased from the control to the intervention period. In a similar study by the authors, 32 Danish participants (15 males; mean age 26 years, 17 females; mean age 65 years) with normal weight were exposed to sunscreen creams with the added chemical UV absorber benzophenone-3 for a 5-day period [61]. Urinary concentration was below the limit of detection (LOD) at baseline and increased following application of the benzophenone-3 added cream among both females and males.

Three studies examined only triclosan exposure. A randomized intervention was nested within a US birth cohort where mothers were provided with wash products (toothpaste, dishwashing liquid, liquid and bar soap) either containing triclosan (78 females in the triclosan arm) or without (76 females in non-triclosan arm) [58]. Over the intervention period, an increase was observed in the triclosan arm compared to the non-triclosan arm. Similarly, a study in Sweden among 12 adults (5 males, 7 females) which instructed participants to use toothpaste containing triclosan for a period of 14 days found increases in triclosan levels [57]. Finally, a double-blind randomized cross-over study in the US provided 16 participants (11 females, 5 males, average age of 43 years) with toothpaste, soap, and dish soap with or without triclosan [63]. After a washout period of at least 16 days where participants were requested to remove all triclosan-containing products from their daily use, participants were randomized to one condition for 4 months and then switched to the other condition for a subsequent 4 months. Compared to baseline, triclosan concentrations increased at the end of the triclosan-containing phase and decreased at the end of a non-triclosan phase.

### Interventions on Dietary Intake or Food Packaging

Twelve intervention studies only altered exposures from dietary intake or food packaging with the majority of interventions targeting BPA [48, 49, 51–53, 64–67]; only two also reported concentrations of other bisphenols such as bisphenol S (BPS) or F (BPF) [45, 67] (Table 2). Few studied other phenols; one study targeted triclosan [64], two benzophenones or parabens [50, 64]) while five studies targeted phthalates [50, 54, 65, 66]. Most studies were conducted in the US [52, 54, 64–66] or in Taiwan or South Korea [49–51, 53, 55, 67] and only one relied on a European population in England [48]. Most studies aimed at reducing exposure through removal or replacement of dietary intake or food packaging [50, 54, 55, 65, 66] or through an educational intervention [48, 51, 67]. Four studies aimed to increase exposures to the chemicals of interest by providing the participants with food or beverage in containers likely to contain the exposures [49, 52, 53, 64] such as canned foods and polycarbonate bottles known to contain BPA; studies would provide participants with bottles to use or canned foods or drinks during the intervention period to understand whether urinary concentrations increased following their use/consumption. Interventions lasted from 3 days [54, 65, 67] to 4 months [51]. Studies reported results generally in the expected direction with educational interventions generally successful in reducing exposures, though findings from studies reducing or replacing exposures were more mixed as a result of participant compliance, motivation, and contamination, highlighting the difficulties of finding alternatives free of the EDC of interest. Studies providing exposures were successful in increasing biomarker concentrations of the EDC of interest.

#### Interventions Aiming to Reduce Exposures

Five studies aimed to reduce exposures from dietary intake or food packaging [50, 54, 55, 65, 66] (Table 2). In an intervention carried out among 5 families (10 adults, 10 children) living in the US, researchers replaced meals for 3 days [65]. Meals were prepared from fresh and organic ingredients and stored in glass containers with BPA-free plastic lids. Participants also received stainless steel water bottles and lunch containers to prevent contamination from other sources of BPA and phthalates. This intervention led to a statistically significant reduction in urinary BPA and di(2-ethylhexyl) phthalate (DEHP) metabolites, but this decrease was not observed for the other phthalate metabolites monitored (MEP, MBP, monobenzyl phthalate [MBZP], monomethyl phthalate [MMEP]). In a similar study providing participants (10 females; mean age: 26.4 years) with meals, no reductions were observed in any of the 11 investigated urinary phthalate metabolite concentrations following the 3-day intervention [54]. In the final meal replacement study, Sathyanarayana et al. (2013) randomized 10 American families in a 5-day trial. Researchers replaced participant dietary intakes with fresh, local, and organic foods in the intervention arm or participants were provided with educational materials on reducing exposure in the control arm. Participants in both arms were provided with, and asked to consume their foods using, plastic-free utensils and dishes and to store foods in glass containers. No changes in urinary BPA or ΣDEHP concentrations were observed in the control arm while the intervention arm saw increases in BPA and ΣDEHP concentrations, which dropped post-intervention. These results were later identified to have occurred due to contamination of the foods provided to the participants in the intervention arm.

Two studies went beyond providing replacement meals. A study in Korea involved a 5-day stay at a temple and required participants (*n* = 25; 9 females, 16 males, between 13 and 64 years old) to follow the daily routines of Buddhist monks and a strict vegetarian diet. Creatinine-adjusted urinary concentrations of MEP, MiBP, MnBP, 5-oxo-MEHP, and 5-OH-MEHP were all lower after the intervention period [55]. In another study relying on the same population, the authors found no statistically significant change in urinary concentrations of methylparaben, propylparaben, butylparaben, benzophenone-1, and benzophenone-3 while concentrations of ethylparaben increased [50].

#### Interventions Targeting Knowledge and Behavior-Change

Education-only intervention studies (studies providing education and training about how to avoid products containing chemicals of interest) targeted BPA and BPS [48, 51, 67] (Table 2). A 3-day intervention among 37 Korean families asked participants to refrain from consuming foods and beverages packaged in cans or plastic [67]. Urinary BPA concentrations decreased for both mothers and children while urinary BPS concentrations decreased among mothers only. A longer-term study among 30 females aged 21–27 years examined the effect of an intervention composed of (1) education on potential health effects and how to avoid exposures, (2) monitoring, and (3) peer support via social media [51]. Urinary BPA concentrations significantly decreased from baseline at the first and second follow-up months but not at the third follow-up month [51]. Null results were also reported: in a study among British adolescents (41 males and 63 females aged 17–19 years) where researchers and participants co-designed a set of instructions on how to reduce exposure to BPA from dietary intakes, there was no decrease in urinary BPA following the 7-day intervention period despite good adherence to the intervention by the participants [48].

#### Interventions Aiming to Increase Exposures

Four studies aimed at increasing exposure to BPA [49, 52, 53, 64] (Table 2). Three consisted of providing food or drink from cans [49, 52, 53] while the last provided polycarbonate drinking bottles to the participants [64]. All reported increases in urinary concentrations of BPA. One study utilized a randomized cross-over design [49]. Among 20 volunteers aged 21 to 32 years in Taiwan, participants in the 4-day intervention were first instructed to refrain from canned and plastic-packaged foods. On the second day, participants were randomly assigned to either receive fresh or canned foods for breakfast; following a 1-day wash-out period, participants received the other breakfast condition. Urine samples were collected at set intervals after eating. Urinary BPA concentrations increased when canned foods were consumed with the strongest increase observed 4 h after ingestion compared to when fresh foods were consumed for breakfast. Similarly, a randomized cross-over study with 75 adults (68% female; mean age: 27 years) involved a 5-day period where half of the participants consumed soup for lunch prepared without canned ingredients while the other half consumed canned soup [52]. Following a 2-day wash-out period, participants experienced the other condition. BPA concentrations were higher after canned soup consumption compared to after consumption of soup prepared without canned ingredients. Another randomized cross-over intervention involved 60 elderly adults (93% female; mean age: 73.1 years) in South Korea over three separate visits where two servings of soy milk were provided either in two glass bottles, two cans, or a glass bottle and a can [53]. Each visit was followed by a wash-out period of at least 1 week. Urinary concentrations of BPA were higher after participants consumed both servings in cans and when one serving was canned and the other was bottled compared to when both servings were bottled. Finally, a study carried out in the US among 77 participants aged 18–23 years instructed participants to use polycarbonate bottles for consuming cold beverages over a 7-day intervention period and observed increases in urinary concentrations BPA [64].

### Interventions on PCPs and Dietary Intake or Food Packaging

Six studies intervened on exposures from a combination of PCPs, cosmetics, dietary intake, and food and drink packaging [44–47, 56, 68] (Table 3). Three of these studies targeted BPA [44, 45, 68] and were conducted in Western countries with the remaining three studies targeting phthalates and were conducted in Taiwan, South Korea, and China [46, 47, 56]. Two studies aimed to reduce exposures using a combination of educational intervention materials along with replacement of products such as food and beverage containers and PCPs [44, 45]. Four studies used an education-only approach to reduce exposures by providing information to participants on EDC and their sources as well as recommendations on how to avoid exposures [46, 47, 56, 68]. Studies lasted from 7 days [46] through the length of pregnancy [56, 68] and studies and their approaches were mostly successful in reducing biomarker concentrations of the EDC of interest.

#### Interventions Aiming to Reduce Exposures

Two studies aimed to reduce exposures through replacement of PCPs, dietary intake, and food packaging [44, 45] (Table 3). In a US study, 24 college-aged women (mean age: 20.9 years) with normal weight were randomized to a control or intervention group for a 3-week period [44]. The control group received weekly newsletters which provided information about healthy diet, physical activity, and general information about BPA. Women in the intervention group received the same educational materials as the control group as well as weekly education, feedback, self-monitoring, and positive reinforcement sessions and were also provided with BPA-free products (including food storage containers, water bottles, PCPs, cosmetics, and feminine products) over the duration of the intervention. BPA urinary concentrations decreased in the intervention group compared to the control group. In a follow-up study, the same intervention was repeated among women with obesity and urinary concentrations of analogs to BPA were also assessed. The authors observed a decrease in urinary bisphenol S (BPS) concentrations among those in the intervention compared to control while BPA and bisphenol F (BPF) concentrations did not change [45].

#### Interventions Targeting Knowledge and Behavior-Change

Four studies used educational approaches to decrease exposures from PCPs, dietary intake, and food packaging [46, 47, 56, 68] (Table 3). A Taiwanese study was conducted with 30 girls aged 4–13 years old who were requested to change or refrain from certain behaviors such as handwashing, the use of plastic materials and packaging, microwaved food, and PCPs and cosmetics for 7 days [46]. Compliance to these changes was monitored using a questionnaire that recorded frequency of the behavior and the number of products used; 7 children were found to be completely non-compliant. When examining all participants, only mono-benzyl phthalate showed a change. When only compliant participants were examined, the study found a reduction in all eight measured urinary phthalates concentrations. Another study with 35 pregnant women in China provided participants with written recommendations to alter their diet, including restricting consumption of canned and microwaved food, increase consumption of organic foods, decrease their use of PCPs, and reduce exposure to second-hand smoke and touching materials such as flooring; these recommendations were provided at three time points during pregnancy [56]. Urinary concentrations of all phthalates metabolites decreased from the 1st trimester baseline visit to the 9th month of pregnancy with the exception of MBzP.

A similar but web-based behavioral education intervention on dietary habits, PCPs, and health to reduce exposure to phthalates, BPA, triclosan, and parabens was carried out in South Korea among women (mean age: control 35.1 years; intervention: 35.8 years) over a period of a month [47]. Participants in the control group were sent written information by mail on how to identify EDC of interest, their health effects, and methods for reducing exposure. Participants in the intervention group were provided with access to a web-based program which also provided information on the health effects of the EDC of interest and ways of reducing exposure through diet, food and drink packaging, and PCPs. They additionally had access to a game to identify items within the home which contained the EDC of interest and a search for local facilities which would facilitate exercise in order to help release the EDC of interest. Compared to the control group, women receiving the intervention showed statistically significant decreases in concentrations of MEHP, MEOHP, BPA, methylparaben, ethylparaben, and propylparaben while no statistically significant changes in MEHHP or triclosan were observed. Finally, a study among 230 pregnant French women with a control (women only received information leaflets) and intervention group (women received information leaflets and workshops on indoor air quality, diet, and PCPs) did not show a decrease in urinary concentrations of BPA or parabens [68].

## Discussion

We found evidence that a variety of interventions are able to alter exposure to phenols, phthalates, and parabens commonly found in PCPs and dietary intake and packaging. All interventions that aimed to increase body burdens (e.g., by providing PCPs containing triclosan, polycarbonate bottles, or meals composed of canned food likely to release BPA) found subsequent increases in urinary concentrations of the targeted chemicals. Conversely, studies which removed or replaced these sources of exposure generally observed a decrease in biomarker concentrations. The magnitude of decrease suggests that PCPs, dietary intake, and packaging can substantially impact our daily exposure [44, 59, 60, 65, 69]. These interventions show that it is feasible for individuals to reduce their exposure in a short timeframe and in practicable ways, including scrutinizing labels of PCPs and cosmetics and choosing products that do not contain chemicals of interest [59, 60, 69], or replacing plastic food and beverage storage containers with those made of glass or stainless steel [53, 64]. Overall, interventions that only focused on providing advice and knowledge to the participants were largely successful in enacting change [47, 51, 56, 67]. This suggests that actionable steps can be implemented, such as actively identifying and replacing products used on a daily basis and would have a chance at decreasing exposures but would require participants taking initiative to alter their exposures.

We were not able to find any studies on glycol ethers even though they are commonly present in cosmetics, especially 2-phenoxyethanol as a preservative in PCPs [70]. Biomonitoring surveys [71–73] reported, for example, that phenoxyacetic acid (PhAA), the metabolite of 2-phenoxyethanol, was present in more than 90% of the participants and was correlated with self-reported use of cosmetics [73, 74]; therefore, further intervention studies should consider these chemicals. Given their suspected effects on human health [23, 72, 75–77], decreasing their exposure would likely benefit public health.

Of the 26 identified interventions, BPA and phthalate metabolites were the most targeted chemicals which may be due to the prominent media focus they have garnered. Dietary intake and packaging interventions were more likely to target BPA and be successful either by itself [49, 51–53, 64, 65, 67] or combined with interventions on PCPs and cosmetics [44–46]. Similarly, decreases in phthalates were observed in interventions which involved either introducing or limiting utensils or packaging containing the chemical of interest [49, 52, 53, 64, 65]. Other compounds such as bisphenols other than BPA, UV filters, and parabens were less well-studied by comparison. Interventions which removed and replaced specific sources of exposures such as PCPs were largely successful in decreasing exposures to phthalates [60], parabens [60, 69], triclosan [60], and benzophenone-3 [60] while studies introducing exposures were capable of increasing concentrations of triclosan [57, 58, 63], UV filters [61], phthalates, and parabens [62]. Dietary interventions which removed or replaced food and packaging exposures were also largely successful in decreasing exposures to BPA [65] and phthalates [50, 65] while studies examining the impact of introducing exposures such as through canned foods or polycarbonate bottles were able to increase biomarker concentrations of BPA [49, 52, 53, 64] and benzophenone-3 [64]. Studies targeting removal and replacement of both PCPs and diet and food packaging were capable of reducing biomarker concentrations of several bisphenols [44, 45] and phthalates [46]. Studies focused on education-only interventions were capable of reducing biomarker concentrations of BPA and its alternatives [47, 51, 67], phthalates [47, 56], and parabens [47].

There were some unexpected results with some PCPs studies finding no changes or even increased concentrations in certain metabolites over the intervention period [47, 60, 69]. The only PCPs study assessing BPA found no change in urinary concentrations [69], suggesting that PCPs use is not a major source of exposure for this chemical. In another PCPs study focusing on phthalates, parabens, triclosan, and benzophenone-3 [60], the majority of urinary concentrations decreased in the intervention with the exception of butyl- and ethylparabens; however, these results should be interpreted cautiously since these two compounds were not detected in almost half of the urine samples. In the educational intervention study by Kim et al. (2021) [47], the authors observed no changes in triclosan concentrations. This may be a result of the included recommendations on PCPs focusing on avoiding strongly scented PCPs, which are relevant for phthalates but not for triclosan, a biocide found in toothpaste and mouthwash, and which were not being targeted as part of their recommendations. Several interventions targeting dietary intake did not have the intended impact [48, 50, 54, 66]. In some, such as Sathyanarayana et al. (2013) [66], unexpected results were a result of known contamination, while others [50, 54] could only speculate about contamination as authors were unable to test the replacement products or did not request participants refrain from certain behaviors, such as using plastic cutlery. Other potential contributors to unexpected findings include contamination from replacement products or unintentional exposure from other products that were not replaced during the intervention such as medications, food, and scented products like cleaning products, fabric softeners, and air fresheners [78–81].

These studies highlighted the difficulties of identifying “safer” replacement products. As suggested by Harley et al. (2016) [60], unintentional contamination from the replacement products may have occurred since they were selected based on the ingredient list. Only one study determined the concentration of their target analyte in their products [59] while the others relied on ingredient lists. Similarly, Galloway et al. (2017) [48] co-designed a set of guidelines with their participants on how to reduce their BPA intake but, even with high compliance, urinary levels did not decrease over the intervention. The participants reported that the widespread use of BPA in foodstuff and inadequate labelling of food and food packaging made it difficult to identify BPA-free food and follow the guidelines for a prolonged period of time.

Other unexpected outcomes may be related to low baseline concentrations [45], suggesting that interventions may be most effective among those with higher average levels. Hagobian et al. (2021) [45] observed only decreases in BPS from their dietary intervention and no change in BPA or BPF concentrations. As 50% and 47% of participants at baseline had non-detectable BPA and BPF (compared to 0.04% for BPS), it was difficult to conclude if the intervention was not effective for these compounds or if the study did not have enough power to detect a change.

We found that compliance, motivation, and ease of adopting changes were important factors for success of these interventions. Chen et al. (2015) [46] found it to be a key factor, as decreases in phthalates metabolites were only observed among girls who were compliant. Similarly, interviews with participants after an intervention where all food was prepared and packaged for the participants but which resulted in no decrease in metabolites found that half of the participants were dissatisfied with the provided foods and were not fully compliant [54]. Other barriers noted included the cost and difficulty of purchasing and preparing fresh food as well as the inconvenience of using glass, rather than plastic, storage containers. These examples illustrate not only the importance of understanding acceptability and ease of intervention elements but also that even with a motivated population, it can be difficult to reduce exposures.

The ubiquity of these chemicals and the lack of consistent and clear labelling means that individual behavior change may not have a consistent impact. Chemicals can transfer from food packaging materials and this is influenced by numerous factors such as packaging size, thickness of the contact layer, storage, and temperature [26]. While interventions to reduce and substitute these types and sources can reduce exposures to chemicals, it may not completely eliminate dietary exposure since food contamination can occur in the pre-market preparation and packaging of foods, such as from the migration of phthalates into milk due to the use of tubing in the dairy milking process, and contamination of water and food sources [25, 82]. Labelling and contamination are also concerns for individuals attempting to replace PCPs and cosmetics. Even among compliant participants, Harley et al. (2016) [60] found that, while measured levels of the EDC of interest among participants decreased on average during the PCPs intervention, some participants also saw increases in their exposure to butylparaben, ethylparaben, and MBP, MiBP, which indicate how difficult it can be to reduce exposures even when willingly. While the identification of replacement PCPs — based on ingredient list information and consumer databases available to consumers — reflects real-world conditions and sources of information, complete elimination is difficult, since such sources of information only consider ingredients with no consideration for the packaging. These examples illustrate not only that it takes personal initiative to search for information and resources but also that personal behavior change may not have the desired outcome if labelling is inadequate or contamination exists. Policy which targets exposures at the source have the potential to have a universal and cumulative impact rather than relying on personal initiative.

We identified several gaps in the literature. Despite small sample sizes and relatively short intervention periods, most studies were able to show changes in biomarker concentrations. Studies varied by the number and type of samples collected, with most studies collecting urines, the reference matrix for short half-life chemicals like phenols and phthalates, two collecting blood samples [57, 61], and one collecting colostrum [68],with variation in their number, frequency, and time of collection within day (which was not always specified). Most studies relied on single spot urine which, given the high within-day variability previously reported for these compounds might be an issue, especially if timing of urine collection systematically differed between pre- and post-intervention. Few studies, mainly cross-over studies, collected multiple spot urines spaced over a period of time in order to assess change [49, 52], while others pooled multiple spot urines (from 2 to 5 samples per pool [63, 67]). Only three studies with limited sample size (N ranged from 8 to 26) collected 24-h urine samples [59, 62, 69].

Five of the six interventions to reduce exposure from PCPs focused on women only [44–46, 59, 60] as they are more likely to use PCPs than men [8, 9]. Most studies did not describe the reasoning behind participant choice, though one study enrolled only men and postmenopausal women as the authors wanted participants to have stable hormone levels [61] while another enrolled girls who participated in a previous study because, in that study, the authors had identified higher concentrations of the EDC of interest and presumed that this knowledge would increase participant compliance to the intervention [46]. Participant age was varied, from children to elderly. Since variations in metabolism are expected by ethnicity, sex, weight, and age, heterogeneity in participant characteristics may explain some differences across studies but our findings still suggest that interventions to reduce exposures can be successful across different population groups. Four studies included pregnant women [54, 56, 58, 68], an important period for the developing fetus. We did not identify interventions which targeted the infant population, who, along with young children are likely to be highly vulnerable to the effects of these exposures and for which higher urinary concentrations have sometimes been reported compared to adults [29, 30]. No studies specifically targeted the pre-conception period, a sensitive window of exposure, although several studies were conducted in women of reproductive age.

Given the short half-life of the chemicals under study, with < 6 h for BPA [83] and < 24 h for phthalates [84], even the shortest interventions (e.g., lasting 2 days) were successful which is encouraging for those motivated to change their behaviors. While compliance during interventions has been identified as a challenge, long-term adherence when education-based interventions are implemented is less well-known. Few studies followed-up participants beyond the immediate post-intervention period [51, 56, 66, 68]. One study which provided an education-only intervention followed participants after the intervention for the duration of three menstrual cycles and found decreases in BPA levels but only until the 2nd cycle in the low adherence group [51]. The lack of long follow-up prevented the evaluation of changes in chemical urinary concentrations on health outcomes, as studies would only have been able to consider biomarkers with levels that changes quickly [57, 58, 63].

While BPA substitutes such as BPS and BPF are likely to be prevalent in products labelled BPA-free, we found only two studies examining them even though studies have suggested similar health concerns as for BPA [85, 86]. Similarly, we did not find any studies on glycol ethers, although decreasing exposures to these compounds could have beneficial effects on health [23, 72, 75–77].

Ten studies were conducted in non-Western countries [46, 47, 49–51, 53, 55, 56, 59, 67] while other studies were mostly in the US with only 1 carried out in the UK, 1 in Sweden, 2 in Denmark, 1 in Belgium, and 1 in France. Consumption habits and regulation vary across countries, which may limit generalizability to other countries. For example, interventions advocating for a decrease in consumption of canned foods would have limited effect in countries where BPA is banned from cans (e.g., France) or among populations where canned food intake is low. However, we believe that this does not prevent overall generalizability of the intervention results because, from the range of participant types across all studies, interventions which provided exposure to participants found expected increases, suggesting that the reverse would be true and that the unexpected intervention results were a result of the aforementioned explanations. Additionally, cross-over studies where the participants served as their own controls avoids many sources of variation that can confound results from non-controlled studies. While decreases observed in urinary concentration are not easily comparable between studies (mainly because of differences in study designs, exposure sources targeted, baseline levels, and population), given the observed impact of interventions addressing only a singular source, it is likely that, effects of interventions targeting more than one source of exposure can be additive.

There are several suggestions for future intervention studies targeting an EDC of interest: (1) increasing diversity of countries and cultures to better understand whether the implemented intervention elicits similar results from places with potentially different regulations and habits; (2) targeting recruitment from the infant and pre-conception periods which have not been examined so far; (3) ensuring that there are control groups or control periods to contrast with the intervention group or intervention period; (4) report in greater detail the biomarker sampling strategy, including the timing and frequency of collection and, if possible, collect repeated samples; (5) test replacement products for the EDC of interest rather than rely on ingredient lists; (6) maximize participant motivation and compliance and collect data to understand how these may impact the results.

## Conclusion

In this scoping review, we identified several interventions which were successful in changing individual exposure to the EDC of interest. Studies removing or replacing personal care products were particularly successful in decreasing exposure to phthalates, parabens, and triclosan while those targeting dietary intake and packaging were successful in decreasing exposure to BPA and phthalates. Given the short half-life of these chemicals, interventions of only a few days were able to decrease urinary concentrations. We believe that these results can help provide information for the general public and health practitioners on how individuals can take action to reduce their exposure. However, and as highlighted by interventions which only provided education about the chemicals, finding relevant consumer information on the presence of the targeted chemicals is not easy. This, in addition to the ubiquitous nature of these chemicals, means that altering one aspect of behavior may not result in a tangible change in body burden. Therefore, policy which targets the use of these chemicals across multiple sectors — in all aspects of processing, manufacturing, and packaging — would have the widest impact with the lowest burden.

## Supplementary Information

Supplementary file1 (DOC 48 kb)

## Abbreviations

5-oxo-MEHP/MEOHP: Mono(2-ethyl-5-oxo-hexyl) phthalate
5-OH-MEHP/MEHHP: Mono(2-ethyl-5-hydroxyhexyl) phthalate
ATHLETE: Advancing Tools for Human Early Lifecourse Exposome Research and Translation
BPA: Bisphenol A
BPF: Bisphenol F
BPS: Bisphenol S
CI: Confidence interval
DEHP: Di(2-ethylhexyl) phthalate
EDC: Endocrine-disrupting compound
EU: European Union
GM: Geometric mean
GSD: Geometric standard deviation
HDAS: Healthcare Databases Advanced Search
LOD: Limit of detection
mL: Milliliter
MCPP: Mono-(3-carboxy-propyl) phthalate
MCNP: Mono-carboxy-isononyl phthalate
MCOP: Mono-carboxy-isooctyl phthalate
MEP: Monoethyl phthalate
MECPP: Mono-(2-ethyl-5-carboxypentyl) phthalate
MEHP: Mono-(2-ethyl-5-hydroxyhexyl) phthalate
MBP: Monobutyl phthalate
MBzP: Monobenzyl phthalate
µg: Microgram
MiBP: Monoisobutyl phthalate
MMeP: Monomethyl phthalate
ng: Nanogram
nmol: Nanomole
PCPs: Personal care product
pg: Picogram
Phaa: Phenoxyacetic acid
SE: Standard error
SG: Specific gravity
TDI: Tolerable daily intake
µL: Microliter
UV: Ultraviolet
US: United States
UK: United Kingdom
