# Supplementary material for: Interventions to Reduce Exposure to Synthetic Phenols and Phthalates from Dietary Intake and Personal Care Products: a Scoping Review
Source: Curr Environ Health Rep. 2023 Mar 29;10(2):184–214. doi: 10.1007/s40572-023-00394-8 (PMC10300154; doi:10.1007/s40572-023-00394-8)
Supplement: Supplementary file 1 — Supplementary file1 (DOC 48 kb) [file 40572_2023_394_MOESM1_ESM.doc]

**Additional file 1**. Search strategy for: (a) PCPs; (b) food and diet. Search was conducted on

Healthcare Databases Advanced Search (HDAS) using the Medline, CINAHL, EMBASE databases.

(a)

| 1. | | (intervention*).ti,ab | | --- | |
| --- | --- | --- |
| 2. | | (personal care product*).ti,ab | | --- | |
| 3. | | (beauty care product*).ti,ab | | --- | |
| 4. | | exp COSMETICS/ | | --- | |
| 5. | | (hair colo?r* OR sun cream* OR sunscreen OR nail polish OR face cream* OR soap* OR conditioner* OR hair dye OR cleansing pads OR colognes OR cotton swabs OR cotton pads OR deodorant OR eye liner OR eyeliner* OR make up OR facial tissues OR lip gloss OR lipstick OR lip balm OR lotion OR makeup OR hand soap OR facial cleanser OR body wash OR nail salon OR pomade OR perfume* OR shaving cream OR moisturizer OR moisturiser OR talcum powder OR toilet paper OR toothpaste OR facial treatments OR wet wipes OR shampoo OR hand saniti?er* OR hand disinfectant* OR skin care product OR cleaning product* OR household product*).ti,ab | | --- | |
| 6. | | (phthalate* OR paraben* OR triclosan OR benzophenone OR acetone OR volatile organic compound* OR allergen* OR endocrine disruptor* OR bisphenol OR BPA OR BPS OR triclocarban OR anti?bacterial OR fragrance* OR UV filter* OR ultra?violet filter* OR glycol ether* OR phenoxyethanol OR ethanolamine* OR dichlorobenzene OR non-ionic surfactant* OR tea tree oil* OR lavender OR stain-resistant OR vinyl OR petroleum-based OR environmental toxin* OR ethylene glycol phenyl ether OR Ethoxyethoxyethanol OR diethylene glycol ethyl ether OR Butoxyethanol OR ethylene glycol butyl ether OR Ethoxybutoxyethanol OR diethylene glycol butylether).ti,ab | | --- | |
| 7. | | (beauty OR cosmetic*).ti,ab | | --- | |
| 8. | | (6 AND 7) | | --- | |
| 9. | | (2 OR 3 OR 4 OR 5 OR 8) | | --- | |
| 10. | | (exposure*).ti,ab | | --- | |
| 11. | | (1 AND 9 AND 10) | | --- | |

(b)

| 1. | | (intervention*).ti,ab | | --- | |
| --- | --- | --- |
| 2. | | (diet* OR food*).ti,ab | | --- | |
| 3. | | (food packaging OR food container* OR can* OR tin* OR plastic OR card*).ti,ab | | --- | |
| 4. | | (phthalate* OR paraben* OR triclosan OR benzophenone OR acetone OR volatile organic compound* OR allergen* OR endocrine disruptor* OR bisphenol OR BPA OR BPS OR triclocarban OR anti?bacterial OR fragrance* OR UV filter* OR ultra?violet filter* OR glycol ether* OR phenoxyethanol OR ethanolamine* OR dichlorobenzene OR non-ionic surfactant* OR tea tree oil* OR lavender OR stain-resistant OR vinyl OR petroleum-based OR environmental toxin* OR ethylene glycol phenyl ether OR Ethoxyethoxyethanol OR diethylene glycol ethyl ether OR Butoxyethanol OR ethylene glycol butyl ether OR Ethoxybutoxyethanol OR diethylene glycol butylether).ti,ab | | --- | |
| 5. | | (Exposure*).ti,ab | | --- | |
| 6. | | (2 OR 3) | | --- | |
| 7. | | (1 AND 4 AND 5 AND 6) | | --- | |
